# MRI findings of pancreatic intraductal oncocytic papillary neoplasm: three case reports and review of literature

**DOI:** 10.3389/fmed.2025.1650931

**Published:** 2025-09-19

**Authors:** Yunan Xiang, Xiaojun Chen, Xiafei Zhan, Zhihan Yan, Jiangfeng Pan, Xinyu Wang

**Affiliations:** ^1^Department of Radiology, The Second Affiliated Hospital and Yuying Children's Hospital of Wenzhou Medical University, Wenzhou, Zhejiang, China; ^2^Key Laboratory of Structural Malformations in Children of Zhejiang Province, Wenzhou, Zhejiang, China; ^3^Wenzhou Key Laboratory of Structural and Functional Imaging, Wenzhou, Zhejiang, China; ^4^Department of Radiology, Jinhua Municipal Central Hospital, Jinhua, China

**Keywords:** case report, pancreas, intraductal oncocytic papillary neoplasm, tomography, magnetic resonance imaging

## Abstract

**Background:**

Pancreatic intraductal oncocytic papillary neoplasms (IOPNs) are uncommon precancerous lesions frequently mistaken for other tumors because of their similar imaging characteristics. The concept of IOPN was first proposed by Adsay et al. in 1996. Recently, an increasing number of reviews on IOPN have been published, however, literature focusing on its imaging features remains scarce. This study retrospectively analyzed the clinical, imaging, and pathological data of three patients with pathologically confirmed pancreatic IOPN, alongside a review of the latest findings in relevant literature.

**Case presentation:**

We described three cases of IOPN in elderly patients, with a male-to-female ratio of 1:2. Among these cases, only one patient exhibited abdominal pain, while the other two were asymptomatic. MRI revealed clearly defined mixed-signal masses in the pancreatic head or tail in each case, with the solid components showing moderate enhancement progressively on contrast imaging. After undergoing surgical treatment, all three patients showed no significant clinical symptoms or recurrence signs during follow-up evaluations over 4–6 months.

**Conclusion:**

Our article provides preliminary evidence of MRI characteristics in pancreatic IOPN, which are essential for understanding its imaging features and aiding in differential diagnosis.

## Introduction

Intraductal oncocytic papillary neoplasm (IOPN), a rare precancerous pancreatic ductal tumor, remains limited and poorly characterized in imaging literature globally (1). Adsay et al. first outlined it in 1996 as a distinct clinicopathological entity (2). In 2010, WHO guidelines initially classified it as part of intraductal papillary mucinous neoplasms (IPMN), marking a significant evolution in its classification (3). IOPN was formally reclassified as a separated tumor category in the 2019 WHO digestive system tumor classification due to its unique molecular and histological profile (4). Histopathological hallmarks include arborizing papillae lined by mitochondria-rich oncocytes cells. Genetically, IOPNs generally lack the activating mutations in KRAS and GNAS that are commonly seen in other IPMN subtypes. And molecular profiling reveals characteristic PRKACA/PRKACB fusions (3, 5). Due to the rarity of IOPN, relevant literature, particularly radiologic characterization, remains sparse. In order to address this knowledge gap, we present a retrospective analysis of three surgically confirmed IOPN cases in our hospital, integrating clinicopathological data with imaging findings and relevant literature review.

## **Case** presentation

### Case 1

**Figure 1:**
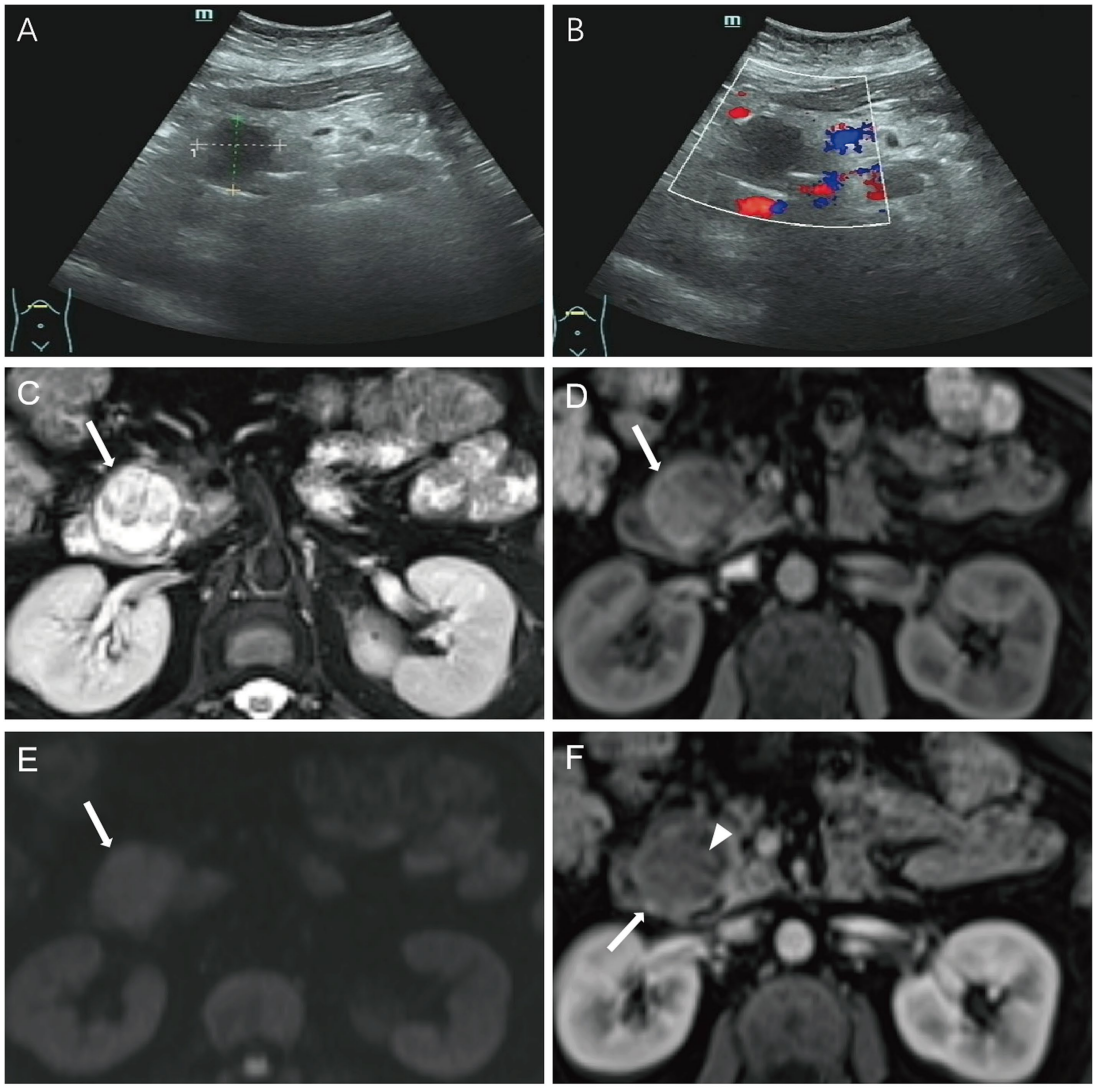
Case 1: abdominal ultrasound (US) revealed a ‌hypoechoic mass lesion anterior to the pancreatic head‌ **(A,B)**. Axial T2 Flair **(C)** and T1WI **(D)** showed a well-defined and mixed signal intensity lesion in the pancreatic head (white arrow). The ‌solid component‌ was hyper-intense on DWI‌ **(E)**. In the arterial phases **(F)**, the lesion demonstrated peripheral enhancement (white arrow), with scattered patchy delayed enhancement internally (arrowhead).

A 61-year-old female presented to a local hospital with symptoms of urinary tract infection. ‌During hospitalization‌, abdominal ultrasound (US) incidentally revealed a ‌hypoechoic mass lesion anterior to the pancreatic head‌ (Figures 1A,B). The patient ‌was subsequently transferred to the general surgery department of our hospital for further treatment of this incidental pancreatic lesion.

MRI revealed a well-circumscribed ‌oval heterogeneous lesion‌ (3.3 × 2.9 cm) in the pancreatic head. The lesion was slightly hypo-intense on T1WI‌ and ‌ hyper-intense on T2WI‌, with internal ‌patchy hyper-intense signals on T1WI (Figures 1C,D). The ‌solid component‌ was hyper-intense on DWI‌ (Figure 1E) and ‌iso-intense to muscle on the ADC map. Post-contrast imaging showed ‌peripheral enhancement‌ of the lesion with ‌patchy delayed enhancement‌ internally. The ‌delayed enhancement intensity‌ of the solid component was ‌slightly lower than that of the normal pancreas‌ (Figure 1F). MRCP (Supplementary Figure 1A) highlighted ‌dilation of the common bile duct and partial intrahepatic bile ducts‌, along with ‌mild dilation of the pancreatic body and tail. The lesion was adjacent to a ‌slightly dilated branch pancreatic duct‌ communicating with the main pancreatic duct.

The mean CT value of the solid portion of the lesion was 42.7 Hounsfield unit (HU) on non-contrast CT, with 71.3 HU in the portal phase and 66.9 HU in the delay period.

Admission blood tests included glucose 9.15 mmol/L, low-density lipoprotein (LDL) 3.58 mmol/L, and carbohydrate antigen 19–9 (CA19-9) 204.91 U/mL. The patient underwent pancreaticoduodenectomy. Gross examination of the pancreas highlighted a 4.5 × 3.6 × 1.8 cm hard and fixed mass with ill-defined borders. Significant postoperative pathological immunohistochemical (IHC) results were as follows: CK7 (−), CgA (+), MUC5AC (+), Muc6 (+), MUC2 (−). The final diagnosis was IOPN with focal microinvasion (approximately 2 mm) ‌and cyst wall nest-like neuroendocrine cell hyperplasia, classified pT1a N0 R0. The patient well recovered without relapse within 6-month follow-up (Supplementary Figures 1B,C).

### Case 2

A 60-year-old female underwent a routine abdominal ultrasound examination that revealed a space-occupying lesion in the pancreatic tail. She subsequently presented to the general surgery department of our hospital in 2024. Dynamic contrast-enhanced CT performed at an external hospital identified a cystic-solid mass in the pancreatic tail, raising suspicion for a neoplastic tumor. Presurgical laboratory tests showed the following results: aspartate aminotransferase 10.9 U/L, LDL 3.77 mmol/L, Albumin 35.7 g/L.

**Figure 2:**
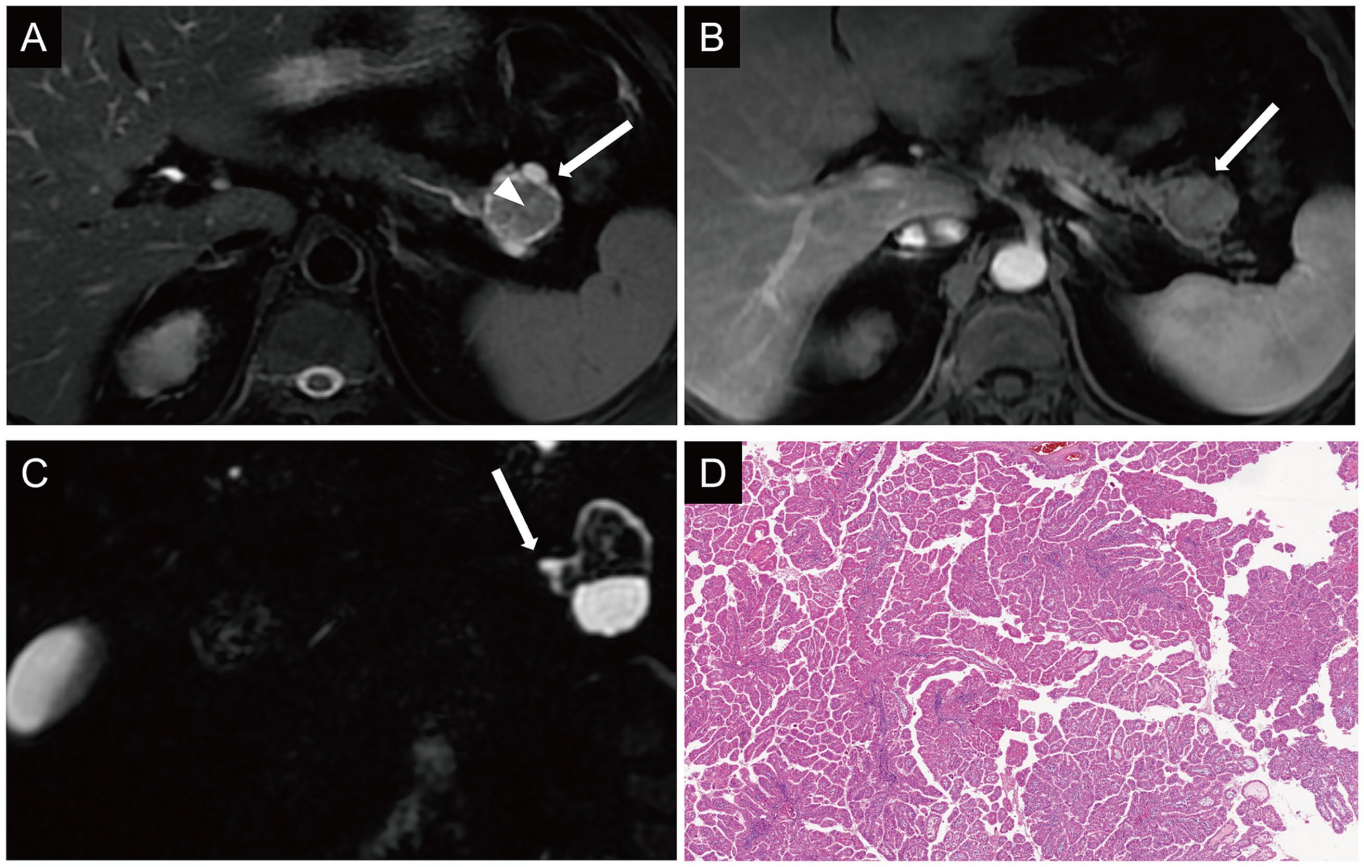
Case 2: axial T2WI **(A)** demonstrated the solid component appeared isointense centrally within the lesion (arrowhead), surrounded by peripheral hyperintense components (arrow). On dynamic contrast-enhanced imaging, the central solid component of the lesion demonstrated enhancement intensity comparable to that of the normal pancreatic parenchyma **(B)**. On MRCP, the cystic component appeared to communicate with a mildly dilated pancreatic duct **(C)**. Histologic diagnosis **(D)** showed IOPN with high-grade intraepithelial neoplasia.

MRI showed a 3.5 × 3.2 cm lesion in the pancreatic hail, which demonstrated mixed isointense to hyperintense signals on T2WI, with iso-intense solid components centrally surrounded by peripheral T2WI hyper-intense areas (Figure 2A). Post-contrast imaging showed the central solid component exhibited enhancement comparable to normal pancreatic parenchyma and the tumor showed persistent enhancement with well-defined margins (Figure 2B). The ‌solid component‌ was hyper-intense on DWI‌ (Supplementary Figure 2A) and ‌iso-intense to muscle on the ADC map. MRCP highlighted the lesion was closely adjacent to the pancreatic duct, and the cystic component appeared to communicate with a mildly dilated pancreatic duct (Figure 2C). No invasion of surrounding vessels or significant lymphadenopathy was observed. Then she underwent distal pancreatectomy and splenectomy. Gross examination (Supplementary Figure 3) showed a solid-cystic tumor located at the tail of the pancreas. IHC results were as follows: MUC2 (−), MUC1 (+), MUC5AC (+++), Muc6 (+++). The after-surgery diagnosis was IOPN with high-grade intraepithelial neoplasia (Figure 2D), classified pT1a N0 R0. In 6-months follow-up, the patient well recovered and did not experience relapse (Supplementary Figure 2B).

### Case 3

A 66-year-old man presented to the general surgery department of our hospital in 2024, complaining of recurrent middle and lower abdominal pain for the past year, with worsening symptoms over the last 3 months. The patient owns personal history of cholangiocarcinoma. Laboratory tests showed no abnormality in CA19-9, cancer antigen 125 (CA125), carcinoembryonic antigen (CEA) and other tumor markers.

Additionally, the mean CT value of the solid portion of the lesion was 36.3 HU on non-contrast CT, with 71.0 HU in the portal phase and 80.5 HU in the delay period.

**Figure 3:**
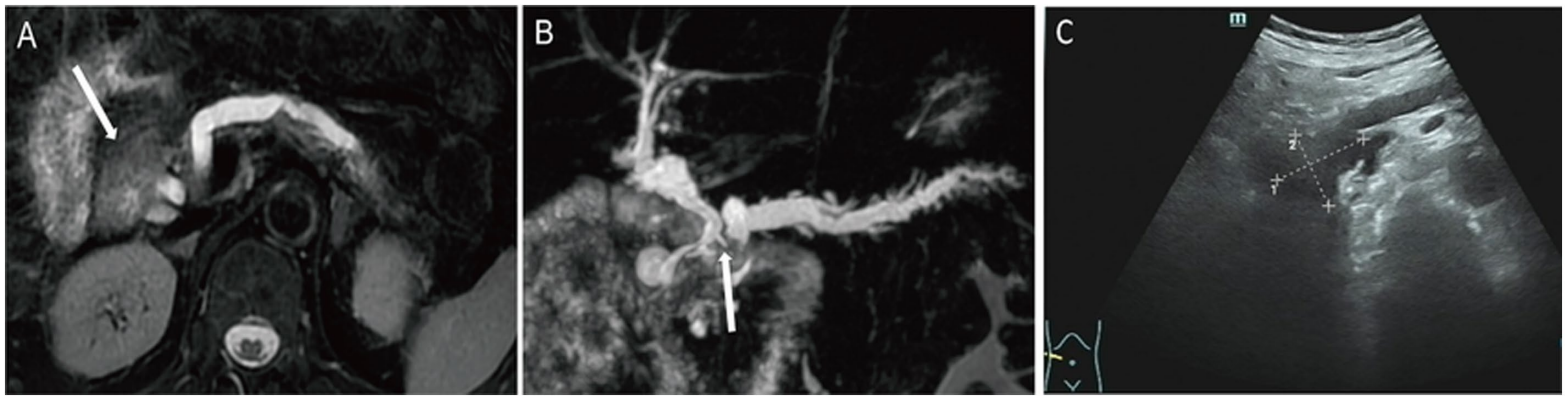
Case 3: axial T2WI **(A)** demonstrated a slightly hyperintense lesion in the pancreatic head, containing internal hypointense septation. On MRCP reconstruction **(B)**, a tumor localized within the main pancreatic duct of the pancreatic head, with marked upstream pancreatic ductal dilation and compression of the common bile duct (white arrow). On US, an anechoic mass **(C)** was seen in the pancreatic head, which communicates with the pancreatic duct.

T2WI demonstrated a mildly hyperintense lesion in the pancreatic head with internal hypo-intense septations and significant dilation of the upstream pancreatic duct (Figure 3A). The lesion was hyper-intense on DWI‌ (Supplementary Figure 4A) and ‌iso-intense to muscle on the ADC map. Post-contrast imaging showed progressive moderate enhancement of the lesion, with enhancement intensity less than that of the normal pancreatic parenchyma (Supplementary Figure 4B). MRCP reconstruction revealed a tumor located within the main pancreatic duct of the pancreatic head, accompanied by significant dilation of the upstream pancreatic duct and compression of the common bile duct (Figure 3B), raising suspicion for a malignant neoplasm of the pancreatic head. The patient underwent pancreaticoduodenectomy. US showed that an anechoic mass was seen in the pancreatic head, communicating with the pancreatic duct (Figure 3C).

**Figure 4:**
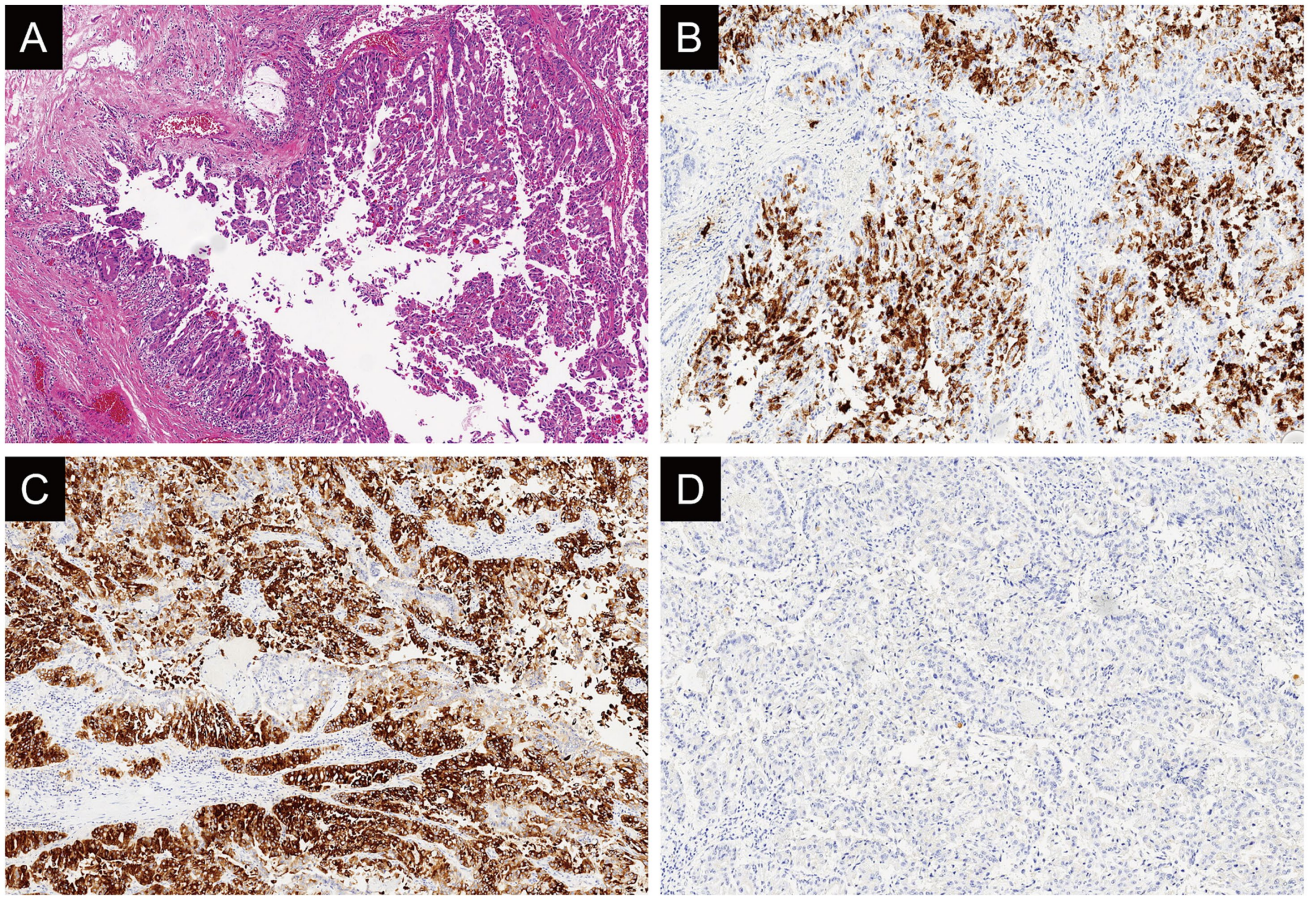
Case 3: histologically **(A)**, the tumor is rich in arborizing papillary architectures composed of oncocytic cells (hematoxylin and eosin stain; original magnification: x4). Immunohistochemically, MUC5AC (**B**: original magnification, x10) and MUC6 (**C**: original magnification, x10) exhibits diffuse and strong expression while MUC2 (**D**: original magnification, x4) is not diffusely expressed in this case.

The final diagnosis was IOPN with high-grade intraepithelial neoplasia and focal moderately differentiated invasive adenocarcinoma (with an invasive focus measuring approximately 3 mm × 1 mm) (Figure 4A). And IHC results (Figures 4B–D and Supplementary Figure 5) were as follows: MUC2 (−), MUC5AC (+), Muc6 (+). CT showed the patient well recovered without relapse within 4-month follow-up (Supplementary Figure 2C).

## Discussion and conclusion

Pancreatic IOPN is a rare digestive system tumor, accounting for 4.5% of pancreatic intraductal neoplasms‌ (6). It was regarded as a pathological subtype of intraductal papillary mucinous neoplasm (IPMN) and has been recognized as a distinct tumor entity in the 2019 WHO classification of digestive system tumors owing to its distinct pathological morphology and genetic mutation (4). To date, fewer than 100 cases of pancreatic IOPN have been reported globally. Wang et al. subsequently published the first systematic review on IOPN, summarizing its comprehensive clinicopathological features (7).

Epidemiological analysis from a systematic review (8) shows a male predominance in IOPNs (male-to-female ratio 1.5:1), with a mean diagnostic age of 58.2 years. Clinically, IOPNs lack pathognomonic manifestations, primarily presenting with nonspecific symptoms, including abdominal pain and jaundice‌. Approximately 34.2% of cases were incidentally detected during routine examinations without overt symptoms, highlighting their indolent biological behavior‌. Importantly, unlike conventional IPMN, serum biomarkers including CEA and CA19-9 show no diagnostic correlation with IOPN progression (normal ranges: CEA < 5 ng/mL, CA19-9 < 37 U/mL) (9).

In our case series, the clinical presentations aligned with established characteristics. The characteristics of these cases are summarized in Table 1. All three patients underwent standardized tumor marker profiling. However, Case 1 exhibited elevated CA19-9 levels (204.91 U/mL), potentially attributed to concurrent biliary obstruction, whereas Case 2 and 3 maintained values within normal limits.

**Table 1:** Patient characteristics.

Variable	Case 1	Case 2	Case 3
Sex	Female	Female	Male
Age (year)	61	60	66
Presurgical laboratory indicators			
Glucose (mmol/L)	9.15 (↑)	5.52	6.43 (↑)
Albumin (g/L)	43.5	35.7 (↓)	33.1 (↓)
ALT (U/L)	26.5	12.9	26.8
AST (U/L)	23.1	10.9	26.6
TBIL (μmol/L)	13.9	12.0	13.4
CA19-9 (U/ml)	204.91 (↑)	13.67	10.22
CA125 (U/ml)	6.46	/	7.23
CEA (ng/ml)	1.98	0.91	1.67
Lesion location	Pancreatic head	Pancreatic tail	Pancreatic head
Largest dimension of lesion (cm)	4.5	3.5	4.5
Chief complains	/	/	Abdominal pain
Type of surgical approach	Laparoscopic	Laparoscopic	Laparoscopic, open
Follow-up duration (months)	5	6	4
Status at follow-up	Alive, recurrence-free	Alive, recurrence-free	Alive, recurrence-free

ALT: Alanine Aminotransferase; AST: Aspartate Aminotransferase; TBIL: Total Bilirubin; CA19-9: Carbohydrate Antigen 19–9; CA125: Cancer Antigen 125; CEA: Carcinoembryonic Antigen. The normal limits of above laboratory data: Glucose (3.9–6.1 mmol/L), Albumin (35–50 g/L), ALT (5-45 U/L), AST (10-40 U/L), TBIL (3.4–20.5 μmol/L), CA19-9 (<37 U/mL), CA125 (<35 U/mL), CEA (<5.0 ng/mL).

Paolino et al. (8) reported the average diameter of IOPNs was 45.5 mm in a comprehensive analysis of 289 cases. These tumors are pathologically characterized by confinement within dilated pancreatic ducts and present with arborizing papillary architectures composed of mitochondria-rich oncocytic cells with large, distinct nucleoli, without obvious mucin production (3). Histopathological evaluation revealed that almost all cases (96.6%) demonstrated high-grade dysplasia, and half of the cases showed invasive carcinoma ‌‌ (8). Moreover, Hirabayashi et al. explained that the presence of hyalinised fibrovascular cores is more commonly described in IOPNs than in IPMNs (10). Molecular profiling further differentiates IOPNs through their unique genetic signature: IOPNs lack certain gene mutations found in IPMN or pancreatic ductal adenocarcinoma, such as KRAS, GNAS, TP53, SMAD4 and CDKN2A (11). Instead, they demonstrate recurrent kinase fusion events, with PRKACB: ATP1B1 being the predominant genetic alteration, followed by PRKACA-related fusions (5, 8). Some scholars regard these gene fusions as driving factors for oncocytic differentiation (12).

As for IHC, almost all IOPNs express MUC5AC, similar to IPMN. MUC1 (EMA) expression is diffusely positive in IOPNs, overlapping with features of ITPNs and pancreatobiliary-type IPMNs (9). Additionally, MUC6 also exhibits diffuse and strong expression, showing tumor pyloric lineage differentiation, although its expression intensity is characteristically weaker than that in ITPNs (8). MUC2‌/CDX2 expression is limited to ‌focal goblet cell components‌, contrasting sharply with the diffuse positivity pattern in intestinal-type IPMNs (3, 8). Moreover, Oncocytic elements exhibit a characteristic granular positivity for HepPar1 (9). Compared with other subtypes of IPMN, CD117 expression is significantly more common in IOPN (13). Research regarding the immune microenvironment has found that IOPNs are enriched in immune cells, with the most notable finding being an increased proportion of CD8 + cells within the infiltrating component. This finding may explain the occurrence of active autoimmune surveillance in invasive IOPNs, which could be associated with the favorable prognosis of IOPN patients (14).

In summary, our findings align with prior reports demonstrating that IOPN exhibits ‌distinctive clinical and pathological characteristics‌. The IHC characteristics of these cases are summarized in Table 2.

**Table 2:** Postoperative IHC results of 3 cases.

Variable	Case 1	Case 2	Case 3
MUC2	−	−	−
MUC5AC	+	+++	+
MUC6	+	+++	+
MUC1	/	+	/
MLH1	+	/	+
MSH2	+	/	+
MSH6	+	/	+
Her-2	−	/	++
P53	+	+	+
P16	/	+	/
Ki-67	+ (2%)	/	+
SMAD4/DPC4	/	+	+
CD56	+	/	/
CD117	/	+++	−
CK7	−	/	/
CgA	+	/	/
Syn	+	/	/
Catenin-β	/	+	/
Hepatocyte	/	+++	+

IHC: immunohistochemical.

In the largest multicenter cohort study to date (*n* = 414), Paolino et al. (8) systematically described the clinical, pathological and radiological features of IOPNs. The pancreatic head (131/237; 55.3%) was the most common site for IOPNs, followed by the tail (33/237; 13.9%) and body (24/237; 10.1%)‌. Main duct and branch duct involvement were equal, at about 38.2%, and the involvement of both the main and branch ducts was 23.6%. In terms of imaging presentation, cystic morphology constituted the majority (24/38; 63.2%), followed by solid lesions (7/38; 18.4%) and mixed cystic-solid architecture (5/38; 13.2%)‌. In our case series, two cases were located in the pancreatic head, one in the body/tail region; two cases were classified as branch-duct type, and one as main-duct type. One solid tumor was localized within the main pancreatic duct of the pancreatic head while the others were cystic-solid tumors with well-defined peripheral encapsulation.

In previous reports (6, 7), large multilocular cystic architecture with MPD dilatation and predominant solid components occupying the majority of the cyst‌ volume were the main characteristics of the imaging of IOPNs. The nodules, thickened septa, or cyst walls were contrast-enhanced because the solid components of the tumor were highly vascularized. According to MRI findings, IOPNs were reported to exhibit predominantly ‌hypointense signals on T1WI and hyperintense signals on T2WI. What is more (9), the characteristic of ‌complex and arborizing papillae‌ suggested a possible diagnosis of IOPN. Additionally, bile duct obstruction and dilatation of the downstream MPD could also be observed (15).

In this case series, the imaging characteristics of the lesions demonstrated consistent patterns across multiple modalities. The lesions predominantly exhibited hypointense signals on T1WI and hyperintense signals on T2WI, which agreed with established radiographic descriptions in the literature. The solid components of the tumors demonstrated moderately progressive enhancement on contrast-enhanced dynamic imaging, with enhancement intensity measuring slightly lower than or comparable to that of the adjacent pancreatic parenchyma. DWI further characterized these solid portions as exhibiting mild diffusion restriction.

Notably, all tumors were radiologically identified within either the MPD or branch pancreatic ducts. Therefore, this anatomical characteristic underscores the critical importance of meticulously evaluating the spatial relationship between the lesions and the pancreatic ductal system was critical for accurate diagnosis. In contrast to extraductal neoplasms, which typically demonstrate mass effect manifesting as ductal displacement or compression, the branch duct-type intraductal tumors presented as cystic-solid lesions that maintained intimate anatomical continuity with the involved duct. These intraductal masses were consistently associated with mild dilation of the affected branch pancreatic duct, which maintained patency at its junction with the MPD.

Studies (16, 17) have shown that an increase in mitochondrial number within oncocytic cells leads to heightened metabolic activity in oncocytic tumors. This pathophysiological mechanism explains the significant 18F-fluorodeoxyglucose (FDG) avidity observed in IOPNs, which demonstrated through positron emission tomography (PET) imaging. What’s more, this metabolic profile frequently results in diagnostic confusion with malignant processes. The current diagnosis relies on fine-needle aspiration (FNA) (18), allowing for histological evaluation of the characteristic oncocytic cells with eosinophilic granular cytoplasm and nuclear features.

Regarding the clinical malignancy of IOPN, firstly, the study by Paolino et al. (8) found that it universally exhibited high-grade dysplasia. In our cases, two cases showed high-grade dysplasia, one of which was accompanied by focal microinvasion. Additionally, perineural and vascular invasion were infrequently reported, with over 90% of patients achieving excellent long-term survival after the operation (8). Although the risk is low, IOPN does have the potential for metastasis and recurrence. The patients who relapsed did not suffer further relapses after complete pancreatectomy (15). In our case series, all patients showed no signs of recurrence on postoperative imaging or in laboratory tests.

Furthermore, CD117 demonstrated a relatively high positivity rate in IOPN and was highly correlated with the PRKACA fusion gene (13). The loss of CD117 expression was often observed in invasive carcinoma components, suggesting its potential role as a marker for malignant transformation. Significant CD117 expression was noted in our Case 2, indicating possible malignant potential. Additionally, Ki-67 demonstrated a significant correlation with the malignancy of pancreatic neoplasms (19). A higher Ki-67 index was associated with greater tumor aggressiveness. In our cases, the Ki-67 index was consistently low, supporting the classification of pancreatic IOPN as a low-grade malignant tumor. Given its malignant potential, surgical resection is the preferred and highly curative treatment. This underscores the importance of accurate preoperative diagnosis to avoid both overtreatment and undertreatment.

The differential diagnosis of IOPNs mainly includes IPMNs, ITPNs, pancreatic neuroendocrine tumor (PanNET), pancreatic ductal adenocarcinoma (PDAC), and mucinous cystic neoplasm. Table 3 summarized the comparative features of IOPN and other cystic-solid lesions of the pancreas.

**Table 3:** Comparative summary of features between IOPN and other pancreatic cystic-solid lesions.

Variable	IOPN	IPMN	ITPN	Oncocytic PanNETs	MCN
Clinical characteristics					
Mean age of onset, y	63	65	56	/	48.6
Common lesion location	pancreatic head	pancreatic head	pancreatic head	pancreatic head, body, or tail	pancreatic body or tail
Common symptom	nonspecific symptoms	Abdominal pain (50–70%), weight loss (44%), recurrent pancreatitis (23%), and jaundice (17%)	Abdominal pain (60–70%), weight loss (40–50%), jaundice (20–30%)	Nonfunctioning tumors: abdominal discomfort, weight loss, and an abdominal mass; Functioning tumors: hypoglycemia from insulinomas, peptic ulcers from gastrinomas and so on	Abdominal pain, an abdominal mass, weight loss and Jaundice
Imaging features					
CT	Cystic, solid or mixed cystic-solid lesions with MPD dilatation, and the solid components are contrast-enhanced	Dilation of the main pancreatic duct, cystic changes in the branch ducts, and a solid nodule. The solid nodule showed mild to moderate enhancement in the arterial phase	Solid or solid-cystic intraductal masses accompanied by upstream pancreatic duct dilation	Intense enhancement in the arterial phase with persistent enhancement in the venous phase	A thick-walled cystic mass that did not communicate with the main pancreatic duct, with calcification of the wall or septa. Enhancement of mural nodules or septa was seen on contrast-enhanced scans
MRI	‌Hypointense signals on T1WI, hyperintense signals on T2WI, and MPD dilatation on MRCP	The cystic fluid appeared hyperintense on T2WI. MRCP clearly demonstrated dilation of the main pancreatic duct and a “cluster of grapes” like change in the branch ducts.	Isointense signals on T1WI, mildly hyperintense signals on T2WI “Two-tone duct sign” and “cork-of-wine-bottle sign” were seen.	Isointense signal on T1WI, mildly hyperintense signal on T2WI, and hyperintense signal on DWI	Malignant lesions exhibited a “honeycomb” or “cluster of grapes” appearance. The cystic fluid appeared hyperintense on T2WI. The main pancreatic duct may be dilated, accompanied by upstream pancreatic atrophy.
EUS	The mural nodule or solid component could be observed.	Intrapancreatic cystic lesion with thin walls and dilation of the main pancreatic duct	Dilation of the main pancreatic duct with a mural nodule	A hypoechoic solid mass with well-defined borders and scattered calcifications within it	Unilocular cystic lesion
Pathological features					
Gross	Intraductal growth with the cystic cavity filled by red, papillary projections and scant mucin	The cyst was filled with mucus and papillary structures were visible.	A grayish-white solid nodule within the pancreatic duct. The cut surface revealed areas of necrosis and fibrosis.	A solid mass	A solitary multilocular cystic mass
Histopathological	Arborizing papillary architectures composed of mitochondria-rich oncocytic cells	Papillary fragments of mucinous epithelium in a background of abundant extracellular mucin	Tubulopapillary structures within the main pancreatic duct, lined by cuboidal to columnar cells with eosinophilic or amphophilic cytoplasm, and virtually no mucin production	Nests of eosinophilic cells with cytoplasm packed with mitochondria	The epithelium was composed of a single layer of columnar mucinous cells, with goblet cells visible in some areas. The ovarian-type stroma consisted of spindle cells and luteinized cells.
IHC	HepPar-1: Diffusely positive CD117: Diffusely positive MUC5AC: Positive (gastric phenotype) MUC1: Focally positive MUC2: Positive only in goblet cells MUC6: Variably positive (diffuse or focal)	MUC5AC: Diffuse positivity (81% of cases) MUC2: Negative MUC1 and MUC6: Positive HepPar-1: Positive - noted in 3 out of 4 cases showing hepatocyte antigen positivity CD117: 94.1% positive	MUC1: 90% positive MUC6: 70% positive CK7/CK19: positive MUC5AC: negative CK20: negative	PDX-1: positive Syn: positive CgA: positive	MUC5AC: 90% positive ER/PR: 85% positive CK7/CK19: positive

Data were adapted from references (7, 8, 15, 20, 21, 29–33).

Similar to IOPN, IPMN is classified into main duct type, branch duct type, and mixed type based on the extent of lesion involvement (15). IPMN secretes abundant mucin with minimal solid components, whereas IOPN typically demonstrates a low degree of mucin production (20). Additionally, IPMN cases exhibit higher frequency of peripheral invasion and worse prognosis than IOPN, reflecting IPMN’s propensity for progression to invasion carcinoma (15).

Both ITPNs and IOPNs typically present as solid or solid-cystic intraductal masses accompanied by upstream pancreatic duct dilation. Distinctively, ITPN classically demonstrates the “two-tone duct sign” and “cork-of-wine-bottle sign.” Histologically, features of ITPN include the absence of eosinophilic cytoplasm and virtually no mucin production (21, 22). And on IHC, MUC5AC is typically negative (23). Compared to IOPNs, previous studies have shown that aggressie tendencies, such as vascular invasion, are more frequently observed in ITPN (21).

Oncocytic PanNETs originate from the pancreatic islet cells and are classified as functional or non-functional based on their ability to produce metabolically active hormones such as insulin, glucagon, vasoactive intestinal peptide, and so on (24, 25). Functional tumors are mostly hypervascular small tumors, with insulinomas and gastrinomas demonstrating marked enhancement in the arterial phase of contrast-enhanced imaging; non-functional tumors are typically larger and often accompanied by necrosis or calcification (25). The enhancement patterns of both entities aid in differential diagnosis.

On cross-sectional imaging, pancreatic ductal adenocarcinoma (PDAC) typically manifests as an irregular, hypovascular mass demonstrating characteristic imaging biomarkers, including pancreatic duct dilatation and parenchymal atrophy, with a demonstrated predilection for perivascular invasion (26)‌. The tumor marker CA19-9 levels are significantly elevated‌ in most cases, serving as a diagnostic adjunct. Current management paradigms emphasize multidisciplinary approaches incorporating neoadjuvant therapy, surgical resection when anatomically feasible,and adjuvant chemotherapy. Despite therapeutic advances, the 5-year survival rate remains dismally low at under 10%, and the disease is prone to locoregional recurrence or distant metastasis ‌ (27).

Mucinous cystic neoplasm (MCNs) predominantly affect postmenopausal women and are commonly located in the body and tail of the pancreas (28). Approximately 4–12% of cases progress to invasive carcinoma. Imaging reveals well-circumscribed multilocular cystic lesions, linear septations, and thickened cyst walls enhancing on contrast phases. High-risk stigmata for malignant transformation include a maximum lesion diameter greater than 4 cm and the presence of mural nodules, solid components, or upstream pancreatic duct dilation (29).

In conclusion, IOPN represents a rare pancreatic entity characterized by distinctive clinicopathological hallmarks. Definitive diagnosis necessitates mulimodal integration of MRI findings, molecular profiling, and immunohistochemical signatures. Accurate identification is critical for guiding surgical planning and predicting favorable prognosis. While this series contributes to the existing literature, the limited cohort underscores the imperative for multicenter registry studies to enhance doctors’ understanding of IOPNs.

## Data Availability

The original contributions presented in the study are included in the article/Supplementary material, further inquiries can be directed to the corresponding author/s.

## Supplementary material

SUPPLEMENTARY FIGURE 1 Case 1: MRCP **(A)** highlighted ‌dilation of the common bile duct and partial intrahepatic bile ducts‌, along with ‌mild dilation of the pancreatic body and tail. The lesion was adjacent to a ‌slightly dilated branch pancreatic duct‌ communicating with the main pancreatic duct. DWI **(B)** and contrast-enhanced sequences **(C)** showed no evidence of significant recurrence.

SUPPLEMENTARY FIGURE 2 The ‌solid component‌ was hyper-intense on DWI‌ of Case 2 **(A)**. Postoperative CT scans for both Case 2 **(B)** and Case 3 **(C)** showed no evidence of significant recurrence.

SUPPLEMENTARY FIGURE 3 Case 2: gross appearance of the surgical specimen (left: anterior view; right: transverse section).

SUPPLEMENTARY FIGURE 4 Case 3: the lesion was hyper-intense on DWI **(A)‌**. Post-contrast imaging showed progressive moderate enhancement of the lesion, with enhancement intensity less than that of the normal pancreatic parenchyma **(B)**.

SUPPLEMENTARY FIGURE 5 Case 3: immunohistochemically, CD117 (**A**: original magnification, x2) and CD10 (**B**: original magnification, x4) are negative. DPC4 (**C**: original magnification, x2), HeP (**D**: original magnification, x2) and Ki-67 (**E**: original magnification, x2) are positive.
