# Supplementary figures and images for: MRI findings of pancreatic intraductal oncocytic papillary neoplasm: three case reports and review of literature
Source: Front Med (Lausanne). 2025 Sep 19;12:1650931. doi: 10.3389/fmed.2025.1650931 (PMC12491326; doi:10.3389/fmed.2025.1650931)

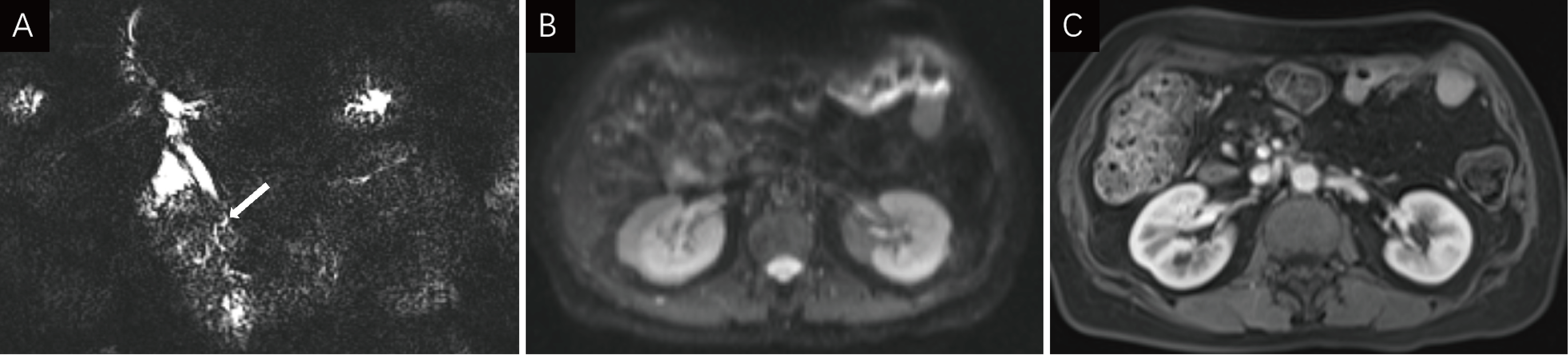

Supplement: SUPPLEMENTARY FIGURE 1 — Case 1: MRCP (A) highlighted ‌dilation of the common bile duct and partial intrahepatic bile ducts‌, along with ‌mild dilation of the pancreatic body and tail. The lesion was adjacent to a ‌slightly dilated branch pancreatic duct‌ communicating with the main pancreatic duct. DWI (B) and contrast-enhanced sequences (C) showed no evidence of significant recurrence. [file Image_1.TIF]

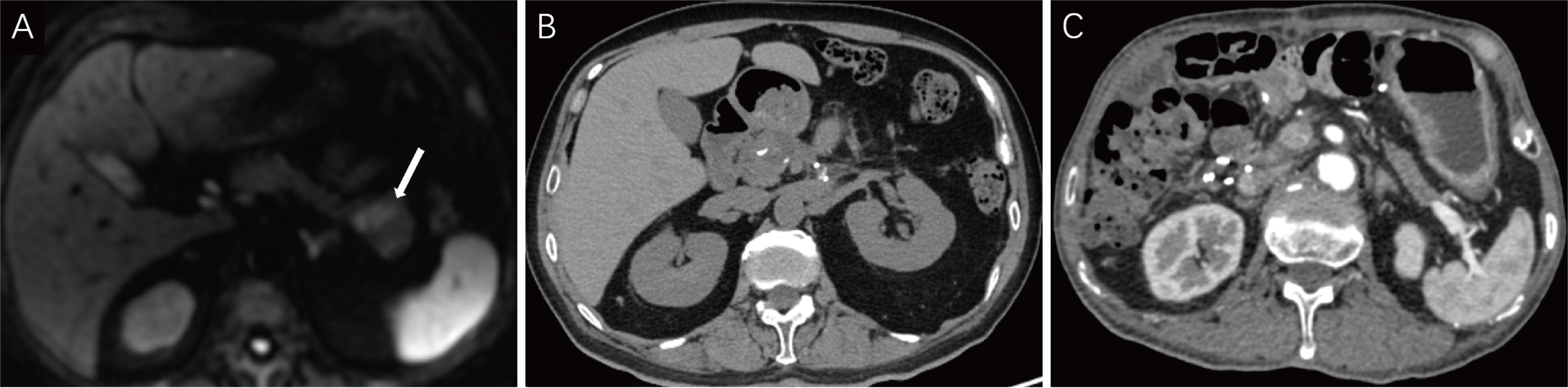

Supplement: SUPPLEMENTARY FIGURE 2 — The ‌solid component‌ was hyper-intense on DWI‌ of Case 2 (A). Postoperative CT scans for both Case 2 (B) and Case 3 (C) showed no evidence of significant recurrence. [file Image_2.TIF]

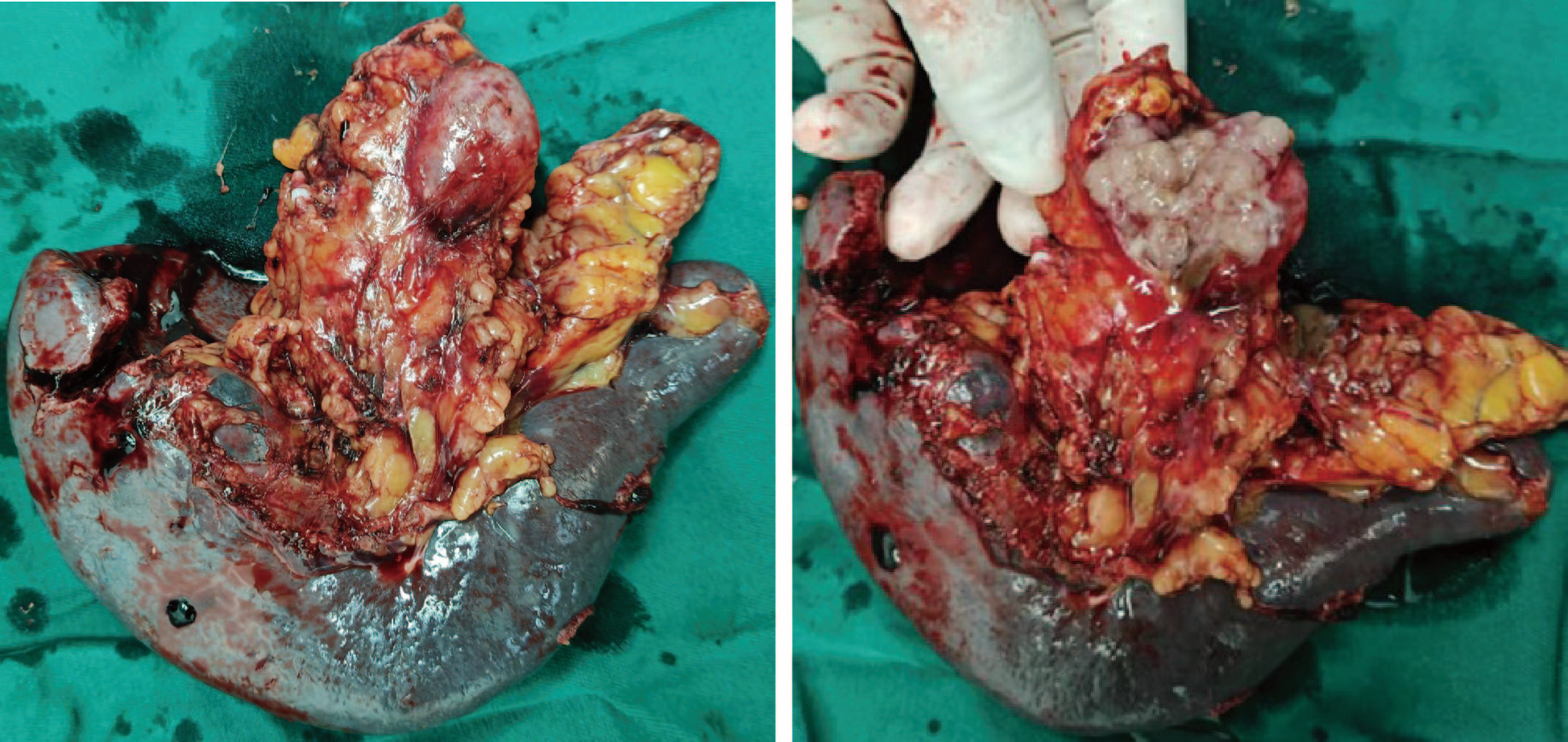

Supplement: SUPPLEMENTARY FIGURE 3 — Case 2: gross appearance of the surgical specimen (left: anterior view; right: transverse section). [file Image_3.TIF]

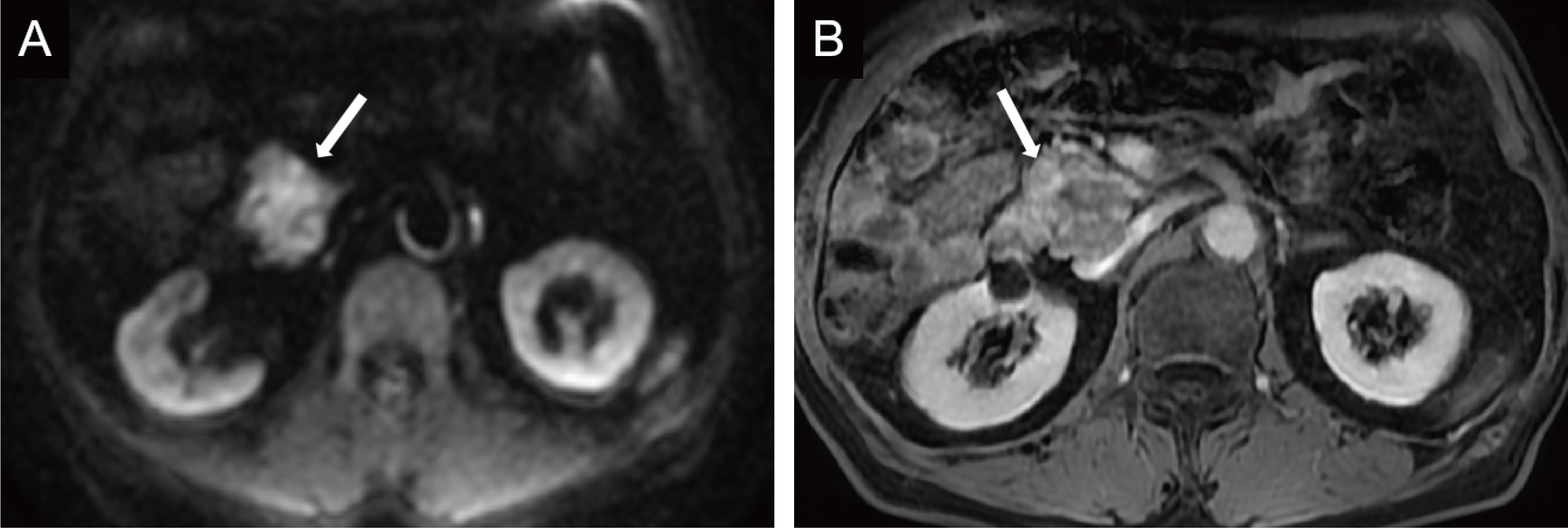

Supplement: SUPPLEMENTARY FIGURE 4 — Case 3: the lesion was hyper-intense on DWI (A)‌. Post-contrast imaging showed progressive moderate enhancement of the lesion, with enhancement intensity less than that of the normal pancreatic parenchyma (B). [file Image_4.TIF]

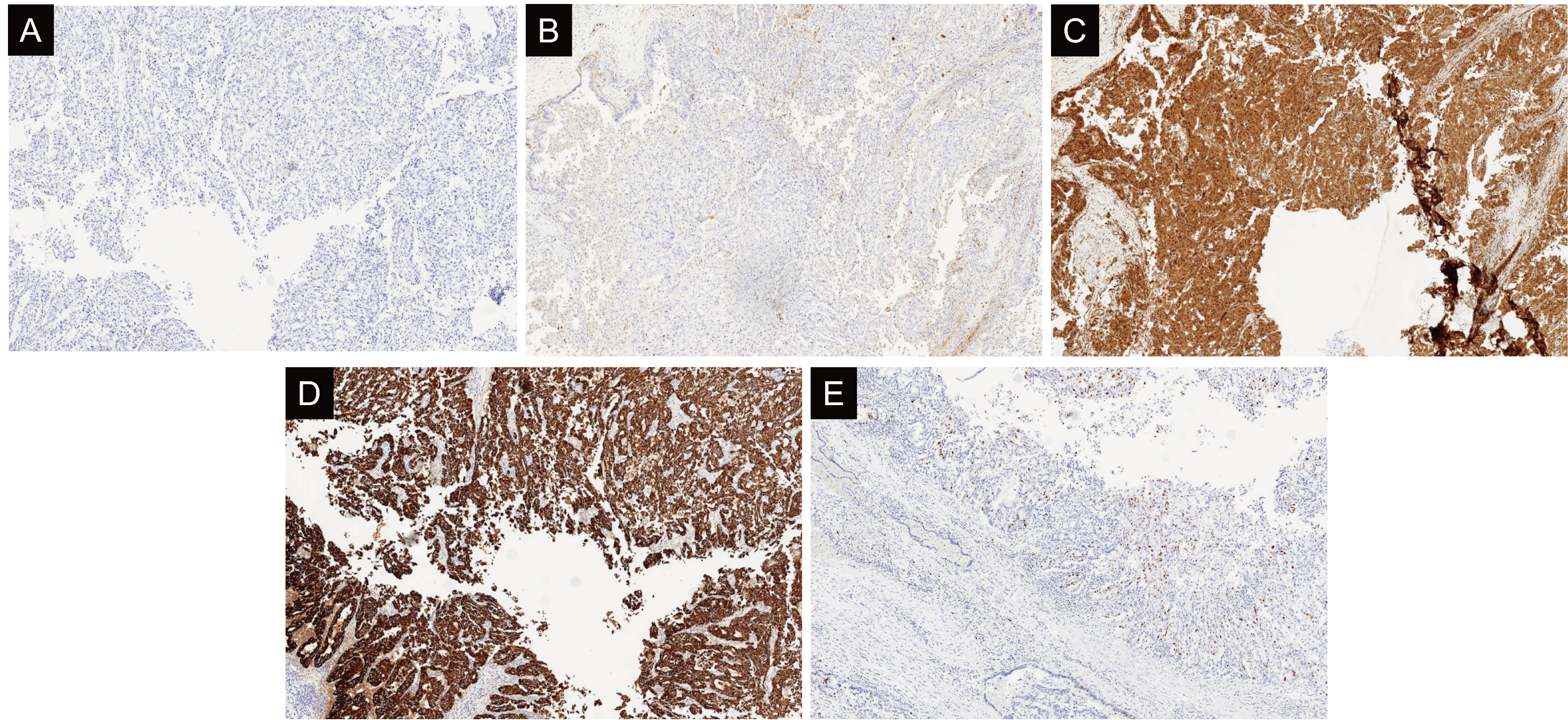

Supplement: SUPPLEMENTARY FIGURE 5 — Case 3: immunohistochemically, CD117 (A: original magnification, x2) and CD10 (B: original magnification, x4) are negative. DPC4 (C: original magnification, x2), HeP (D: original magnification, x2) and Ki-67 (E: original magnification, x2) are positive. [file Image_5.TIF]
